# Genito-Urinary Diseases.—Gonarthritis1Delivered at the Erie county hospital, Buffalo, N. Y., December, 1897.

**Published:** 1898-03

**Authors:** B. H. Daggett, Ross G. Loop


### Clinical Lecture.

### GENITO-URINARY DISEASES.—GONARTHRITIS.[1]

By B. H. DAGGETT, M. D.

Reported by Ross G. Loop, M. D.

THE young man before us today is nineteen years of age, a painter by occupation. There is no family or personal history of rheumatism. About a year ago he acquired gonorrhea. About four weeks after the first appearance of his urethral disease he developed an arthritis affecting the right ankle, and other joints were progressively involved in a lesser degree. He was incapacitated for doing manual labor for five months. Finally the urethral discharge ceased and all the joint symptoms subsided, except induration, thickening and impaired motion of the right ankle.

About the first of December, 1897, a urethral discharge again appeared, whether it was due to a new infection or recrudescence of the old malady I am unable to say. About a week after this development the metatarsophalangeal joints of the second toe of both feet became swollen and tender, followed by renewal of the arthritis in the right ankle and later by involvement of the metacarpophalangeal joints of the middle and index fingers of the left hand. He is pale and anemic. He is undoubtedly suffering from gonorrheal arthritis.

I will give him oil of wintergreen, salol, iron and some bitter vegetable tonic. I also order flushing of the urinary tract with permanganate of potash, 1 part to 8,000—to be followed after two or three days with a solution of ichthyol—tablespoonful to the quart, to be used twice daily. This will probably reduce the urethral irritation to such a degree that weak solutions of some silver salt can be instilled into the deep urethra without any danger of exciting complications. Europhen is also valuable for this purpose. Argonin (full strength) insufflated into the deep urethra, by means of a powder blower, through a tube curved and fenestrated at its distal end is valuable. Mercurial ointment and ichthyol are to be applied to the inflamed joints, which are to be wrapped in flannel rollers. The roller about the ankle joint should be tightly applied in order to promote absorption by mechanical pressure.

1. Delivered at the Erie county hospital, Buffalo, N. Y., December, 1897.

It is stated that gonarthritis is not a common complication of gonorrhea. The records of this hospital indicate that it is more common than is generally supposed. Grissole reports joint involvement in 2.8 per cent. of his cases. Besnier and others allege that it occurs in about 2 per cent. of their cases.

It rarely appears before the fourth week of the urethral inflammation, but it may occur at any time during the existence of specific urethritis at any stage of its existence. Some authorities regard gonarthritis as a complication of posterior urethritis, and state that at this stage of the malady this condition is frequently unrecognised and its causal rôle as an incitor of rheumatism is overlooked.

Gonarthritis may develop from any nidus of infection upon any mucous surface. Many theories have been proposed to explain the etiology of gonarthritis. The discovery of the gonococcus paved the way for the study of its pathology, but its relationship to gonorrhea was recognised long before Neisser (1873) discovered and demonstrated that a specific germ produced gonorrhea.

Rosalimus and Lewin believed that the action of the specific toxin of the gonococcus on the vaso-motors caused the arthritis. Senator alleged that the local irritation affected the trophic nerves. Guyon and Janet affirmed that the ptomaines of the gonococcus caused the metastases. Shuster believed that the combination of gonorrhea and syphilis was accountable for the joint involvement. Diday thought that a rheumatic diathesis must exist before the joint complications occurred, while Theiry denied that any relation existed between the rheumatic diathesis and the joint inflammation occurring in a gonorrheal subject. Peter, Gouillard and others said that it was articular rheumatism, plain and simple. Duboc said it was gout. Loeb said it was due to the gonorrheal process in the posterior urethra. Fernier believed that gonarthritis was due to urethral injury and called it urethral rheumatism. Panas stated that injury to the urethra by catheterisation caused erosion, and infection might induce gonorrheal arthritis. Ricord and Bergh agree with Loeb and consider gonarthritis a complication of posterior urethritis. Bond believed that inflammation of the prostatic veins caused gonarthritis and that inflammation of the lymphatics was never followed by joint complications.

Guerin, Loraine and Lesigne said that gonorrhea was a general disease, with local manifestations, because it caused remote complications. The gonococcus has been found in the joint effusions and in the blood, and hence it is claimed to be the cause of gonarthritis. E. J. Senn alleged that the gonococcus is a pyogenic organism on mucous surfaces, but when latent germs migrate by the blood or lymph and settle upon serous surfaces a plastic inflammation results.

Bumstead said that gonorrheal rheumatism is essentially a hydrarthrosis and that if empyema of the affected joints occurs it is the result of a mixed infection.

The clinical history of gonarthritis is variable: it may be a mild, dropsical condition, readily healed, or a catarrhal condition, resulting in anchylosis, or a severe form, inducing erosion, phlegmon and tenosynovitis or bursitis.

The clinical history, together with the macroscopical and microscopical examination, will give data for diagnosis. Konig said that the urethral secretion should be examined in all cases of catarrhal inflammation of the joints. Fenger said gonarthritis usually attacks several joints. Senn said that it is a monoarticular disease.

This disease spends its force or settles upon one joint. One attack apparently predisposes to another. Recrudescence or a fresh attack of gonorrhea will cause this complication to reappear.

General treatment is antiblennorrhagic and antirheumatic. In the first stages, the local treatment is antiphlogistic, in the latter stages resolvent and absorbent. The treatment is based on its cause, gonorrhea, whether active or chronic. Remove this cause and its complication, gonarthritis, disappears. Never attempt to break up adhesions resulting from gonarthritis until the gonococci have disappeared from the secretions of the urethra as well as from the urine.

The surgery of the graver forms of gonarthritis will be considered at another time.

---

### DYSMENORRHEA.

Dr. H. Talley states (*Phil. Polyclinic*) that a mixture of caffein, potassium bromide and tincture gelsemium is of much value in the treatment of dysmenorrhea. This should be administered for a few days before menstruation.

